# Modifier Role of Common *RET* Variants in Sporadic Medullary Thyroid Carcinoma

**DOI:** 10.3390/ijms222111794

**Published:** 2021-10-30

**Authors:** Anna Skalniak, Małgorzata Trofimiuk-Müldner, Elwira Przybylik-Mazurek, Alicja Hubalewska-Dydejczyk

**Affiliations:** Chair and Department of Endocrinology, Jagiellonian University Medical College Krakow, 30-688 Krakow, Poland; malgorzata.trofimiuk@uj.edu.pl (M.T.-M.); elwira.przybylik-mazurek@uj.edu.pl (E.P.-M.); alahub@cm-uj.krakow.pl (A.H.-D.)

**Keywords:** medullary thyroid carcinoma, *RET* variants, modifier

## Abstract

**Background:** Although the disease-causing effect of pathogenic variants in the gene *RET* has been unambiguously identified, there is a lack of consensus regarding the possible impact of common variants in this gene. Our study aimed to test whether variants in exons 10, 11, and 13–16 that are commonly detected during routine diagnostic testing might have any modifying effect on MTC. **Methods:** In sporadic MTC patients with no pathogenic variants but with or without common variants in *RET*, the following variants were evaluated: rs1799939 (p.G691S), rs1800861 (p.L769=), rs1800862 (p.S836=), rs2472737 in intron 14, and rs1800863 (p.S904=). **Results:** After Bonferroni correction, none of the variants were statistically significantly associated with disease outcome when analysed independently. The MTC group was divided into three genetically different clusters by unsupervised k-means clustering. Those clusters differed significantly in the age at diagnosis. A trend towards the association of given clusters with metabolic disorders and with remission state was identified. **Conclusions:** Although common variants in *RET* are not responsible for the risk of MTC, their analysis might turn out useful in the prediction of a patient’s clinical outcome. Importantly, this analysis would come with no additional cost in laboratories with a diagnostic procedure based on exon sequencing.

## 1. Introduction

Pathogenic germline variants in the protooncogene *RET* are responsible for the hereditary form and part of the apparently sporadic cases of medullary thyroid carcinoma (MTC), and a strong genotype-phenotype correlation has been observed for those variants. Because of the high frequency of hereditary MTC, even among presumed sporadic cases, genetic testing for pathogenic germline variants in the *RET* protooncogene associated with multiple endocrine neoplasia type 2 syndrome (MEN2) should be offered to all MTC patients according to the American Thyroid Association 2015 guidelines [1]. During standard genetic diagnosis of medullary thyroid carcinoma patients (MTC), we observe the repeated appearance of one or more common polymorphisms in the gene *RET*; in the case of sporadic MTC, often with no additionally identified disease-causing variant. Common variants in this gene have repeatedly been shown not to be associated with MTC when analysed individually [2,3,4,5] and are classified as benign or likely benign in databases such as NCBI Clinvar [6] or the Human Gene Mutation Database (HGMD) [7]. Meta-analyses on the association of common *RET* variants with MTC provided conflicting results, showing no significant associations with MTC or identifying some specific associations; however, in most of those studies, no distinction between hereditary and sporadic cases has been made [8,9,10]. Additive effects of *RET* variants or specific haplotypes on MTC risk have been suggested [11,12]. Moreover, it has been argued that common *RET* variants might have a modulatory role in MTC outcome rather than alter significantly the risk of disease development, but also in this aspect, conflicting results have been obtained, as in some studies correlations with clinical parameters were observed, while in others, no variants were significantly associated with the clinical outcome of MTC patients [3,13,14]. The issue of whether *RET* variants classified as non-pathogenic might be modifiers of clinical outcome of MTC has been addressed also in familial MTC cases. For example, an association of intronic variants with lymph node metastases at diagnosis has been noted in *RET* p.Gly533Cys pathogenic variant carriers [15]. In other studies, the presence of common variants correlated with the course of disease in MEN2 patients [16,17]. However, as data are limited, particularly for sporadic MTCs, there is still a lacking consensus regarding the influence of common variants on disease outcome, which may be achieved only as further studies are published.

The aim of our study was to investigate the association of common non-disease-causing *RET* variants with clinical outcome in the population of sporadic medullary thyroid carcinoma patients from southern Poland.

## 2. Results

### 2.1. Association of Individual Variants with Sporadic MTC

A total of 48 patients with sporadic medullary thyroid carcinoma (MTC) with no tumours, signs, or symptoms which may be attributed to MEN syndromes; with no family history of MTC or MEN2 syndrome; and with negative *RET* pathogenic variant status in genetic diagnostics were included in the study. As controls, 48 healthy volunteers were included who were negative for thyroid disorders, had a negative family history for thyroid disorders, and were at least 30 years old at the moment of the study. The gender distribution was similar among the groups: 14 men and 34 women vs. 10 men and 38 women in the MTC and the control group, respectively (*p* = 0.4795).

In order to verify whether the distribution of common polymorphisms in our tested group is in accordance with the general population, we compared the MTC group with healthy volunteers and with literature data.

As expected, the groups did not differ in the minor allele counts nor in the presence or absence of the minor allele of any of the investigated variants, even without the use of any correction for multiple comparisons—all *p*-values for all variants were greater than 0.05. This confirms that the investigated MTC group was not biased in relation to their population of origin.

Minor allele frequencies were comparable to literature data for both groups and did not differ significantly between the groups, even with no applied correction for multiple testing (Table 1).

We observed no deviations from Hardy–Weinberg equilibrium in any of the groups.

The number of patients who were homozygous for all variants was also similar in both groups: 4 and 8 out of 48 in the MTC and control group, respectively (*p* = 0.3553). The two groups also did not differ in the total number of minor alleles (*p* = 0.6642) or number of different minor alleles (*p* = 0.9216) per person.

When analysing our data for haplotypes in Haploview, we observed a strong linkage disequilibrium (LD) between the variants e11 rs1799939 and e15 rs1800863 (r^2^ = 0.964).

Haplotype distribution did not differ significantly between the groups: *p* = 0.7415. The fractions of the identified haplotypes are summarised below (Table 2).

We did also not find any minimal combination of *RET* variants that would discriminate between the both groups, as investigated by step-wise regression analysis (all variables were excluded from the model).

Therefore, common *RET* variants, identified during routine genetics diagnostics and classified clinically as benign, did not differ between patients with MTC and healthy controls in our study group, which was expected and confirms the reliability of our data.

### 2.2. Correlation of RET Variants with Clinical Data

For answering the question of whether any of the variants might have an impact on clinical outcome, we compared clinical data from sporadic MTC patients who were carriers of a given variant with clinical data from sporadic MTC patients in whom none of the variants was present. The results are presented in Table 3 below.

According to the data above, it might be possible that some of the variants might correlate with age at diagnosis or TSH concentrations. However, after the application of Bonferroni correction for multiple comparisons, all results remained insignificant for each of the common *RET* variants analysed independently.

**Figure 1:**
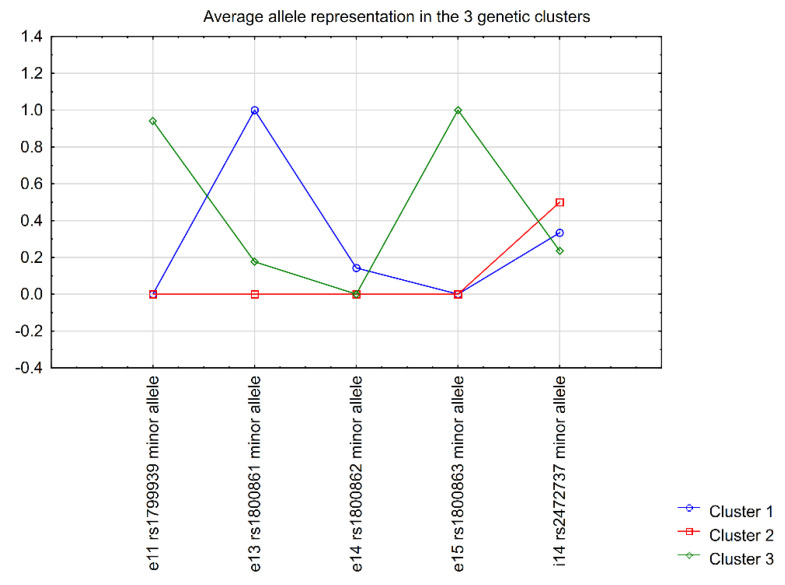
Graphic representation of clusters discriminated by unsupervised k−means clusterisation.

In order to investigate whether sporadic MTC patients with different combinations of common *RET* variants (rather than single variant status) might differ in means of disease outcome, we performed unsupervised k-means clusterisation on the basis of the minor allele status of all investigated variants. For this and all following analyses, only patients from the MTC group were included. The three identified clusters that differed maximally between each other in means of the status of *RET* variants included 21, 10, and 17 patients in clusters 1, 2, and 3, respectively. The graphic representation of the clusters is presented in Figure 1.

The clusterisation divided the MTC group into three clusters that maximally differed genetically between each other on the basis of the five investigated *RET* polymorphisms. Cluster 1 was characterised most significantly by e13 rs1800861 (p.Leu769=) and, to a lesser extent, i14 rs2472737, as well as the rarer e14 rs1800862 (p.Ser836=), which was present solely in patients of cluster 1; cluster 2 was characterised by the absence of minor alleles in all variants besides i14 rs2472737 in some cases; while cluster 3 was characterised by the presence of minor alleles of e11 rs1799939 (p.Gly691Ser) and e15 rs1800863 (p.Ser904=) predominantly (which we found to be in strong LD in our study, as shown above), and i14 rs2472737 in some cases. Generally, i14 rs2472737 was similarly represented in all the clusters and did, therefore, not play a discriminative role in the generation of the clusters.

**Figure 2:**
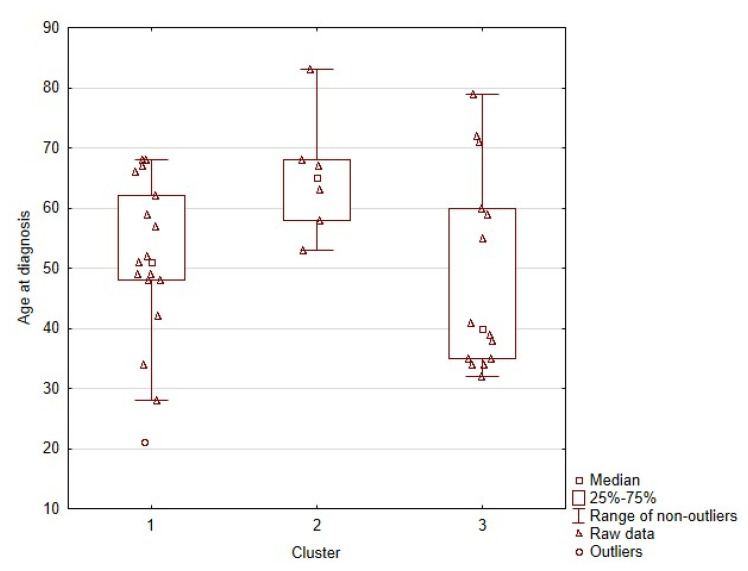
Graphic representation of age distributions in the obtained clusters.

We then analysed whether the obtained clusters differed clinically between each other. Importantly, we found that the groups differed significantly in the age of diagnosis, as shown by Kruskal–Wallis test (*p* = 0.0283). Post hoc analysis revealed that differences between clusters 1 and 2 were responsible for the observed effect, with *p* = 0.0289. The age distribution in clusters 2 and 3 differed visually (Figure 2), but this difference was not statistically significant: *p* = 0.0877.

Cluster 1 differed from cluster 2 mainly in the status of the variant e13 rs1800861 (p.Leu769=). However, when we analysed this variant in the whole patient cohort (independently of the presence of other variants in the patients), it did not correlate significantly with the age at diagnosis: *p* = 0.1051. Therefore, it seems that the absence of any variant besides i14 rs2472737 might be responsible for the observed effect, which would explain the trend observed when analysing the standalone variants independently, in comparison to patients with no variants. When this hypothesis was tested on the whole study sample MTC group, this association, indeed, turned out to be clinically significant, with *p* = 0.0234 and power of 76.67%. MTC patients with no other variant than i14 rs2472737 (in some cases) were on average 65.3 (+/−10.3) years old at diagnosis, in comparison to patients with any other variant present, independently of the status of i14 rs2472737, who were diagnosed at a mean age of 50.1 (+/−15.0) years.

The three clusters did not differ in the stage of the disease at diagnosis of the patients (staging I, II, IIIA/C; according to AJCC, 8th ed. [18]), *p* = 0.7917. The patients did not differ in the frequency of lymph node metastases (*p* = 0.6601), nor in the frequency of distant metastases (*p* = 0.2714).

We were also interested in whether the clusters differed in the presence of other diseases in the affected patients or their families, particularly other cancer types and metabolic disorders. The results are summarised in Table 4.

Therefore, patients classified into the three genetic clusters did not differ in the presence of other tumours and metabolic disorders in themselves or their first-degree family members. However, for metabolic disorders, *p*-values below 0.1 were observed for the patients as well as their family members. According to our results, in order to identify significant differences at α = 0.05 and a power of 80%, a minimal total sample size of 79 and 52 patients would be required in the study for metabolic disorders in the investigated patients and their families, respectively.

**Figure 3:**
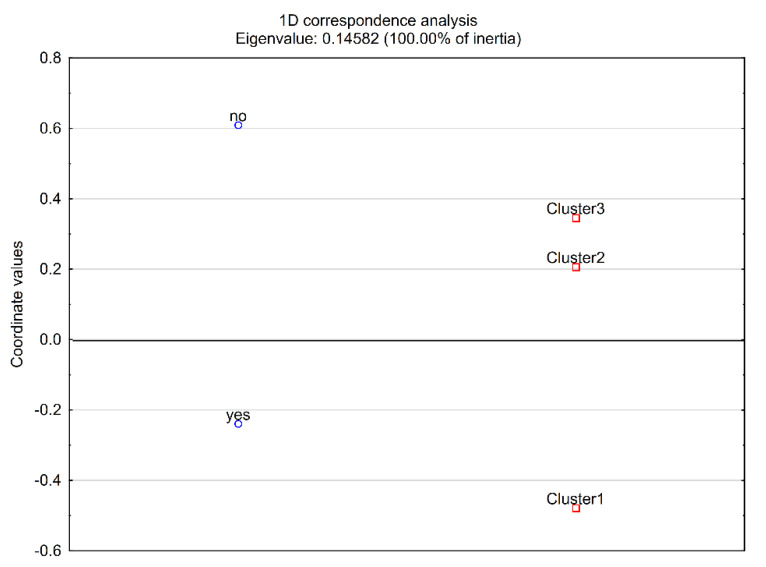
Correspondence analysis plot−relationship between clusters and remission state.

Other clinical parameters were also investigated, although we were aware that at least a part of them (particularly MTC serum markers or TSH levels) may have been subject to modifications during the treatment of the patients. The patient clusters did not differ in relapses (*p* = 0.7475), CEA concentrations (*p* = 0.9887), Ct/proCt (*p* = 0.3164), nor TSH concentrations (*p* = 0.8320). We did, however, observe a difference between the clusters that might reach significance if the tested group was larger, in the remission state (*p* = 0.0582; minimal sample size for *p* < 0.05 and power ≥80%—67 patients), where the presence of remission would be associated with cluster 1, as suggested by correspondence analysis (Figure 3).

Indeed, the decomposition of the CHI^2 table indicated that cluster 1 would differ significantly from clusters 2 + 3 analysed together: *p* = 0.0277 (statistical power: 65.66%), with 14 remission cases and only 1 patient without remission in cluster 1 and 14 remission cases but 10 patients without remission in clusters 2 + 3. In contrast, cluster 2, which was characterised by an older age at diagnosis, would not differ significantly from clusters 1 + 3.

Importantly, neither e13 rs1800861 (p.Leu769=) nor e14 rs1800862 (p.Ser836=), which characterise cluster 1, were associated significantly with remission when analysed independently: *p* = 0.5450 and *p* = 0.1713, respectively. Only when e13 rs1800861 co-occurred with e14 rs1800862 and/or i14 rs2472737 could a tendency towards the correlation with remission state be identified (*p* = 0.0781). However, a larger patient group (n = 78) would be required in order to achieve statistical significance at α = 0.05 with a power of 80%, and additionally, with an appropriate correction for multiple comparisons applied, the minimal patients group would need to be even larger.

## 3. Discussion

In our study group, common *RET* variants did not differ significantly between MTC patients and the control group.

This is in accordance with literature data, where most findings confirm the lack of disease-causing effects of common *RET* variants. Although the authors of some of the studies claimed to have identified significant correlations of given common variants with MTC, their studies included multiple comparisons that were not adjusted for or had a low power of analyses [19,20]. In their study, Kaczmarek-Ryś et al. also analysed the Polish population and found an association of the T allele of p.Leu769= with sporadic MTC when no correction for multiple comparisons was performed [21]. No such association was detected in our database. In contrast, Gemignani et al. showed an association of the G allele of this variant with sporadic MTC [22]; however, they found that the T allele was the minor allele in their group of tested individuals, while in our group, G was the minor allele, which is in agreement with GnomAD, 1000Genomes, TOPMED, and ExAC databases (accessed collectively through the UCSC Genome Browser [23]). This discrepancy might be due to ethnic differences; therefore, the results between populations and associations with diseases may vary. A lately performed meta-analysis showed a significant association of the variant p.Leu769= with the hereditary but not the sporadic form of MTC [24]. This meta-analysis also showed a weak but significant association of p.Gly691Ser and p.Ser904= with sporadic MTC, which was not observed in our study nor in other meta-analyses [2,3,4,5]. The conflicting data, together with weak associations, that would require larger study populations in order to obtain statistical significance at high statistical power, confirm that the impact of common *RET* variants classified as non-disease-causing is very weak, if any, and is therefore not suitable for clinical decision-making about the risk of MTC development.

The standalone effects of the analysed variants as compared to the group of patients with no variants showed a trend of association with age at diagnosis, which is a rough approximation of the age of disease onset. No conclusion could, however, be drawn for any of the variants, as the correction for multiple comparisons rendered all results insignificant.

The comparison of the genetic clusters obtained by unsupervised clusterisation indicated that clusters 1 and 2 differed significantly between each other in age at diagnosis of the patients. Genetically, those two clusters differed between each other predominantly in means of e13 rs1800861 (p.Leu769=); therefore, the difference in age at diagnosis might be associated with this variant. However, it turned out that the variant e13 rs1800861 itself was not significantly associated with the age at diagnosis, which is in agreement with literature data [5]. Another variant, i14 rs2472737, was observed in both clusters to a similar extent. However, cluster 2 is characterised mainly by the fact that no other variant other than i14 rs2472737 was identified in the patients who have been classified into this cluster. Indeed, it turned out that in MTC patients of our study group, the absence of any variant in *RET*, besides i14 rs2472737, was a factor that protected against the development of MTC at a younger age (*p* = 0.0234, statistical power = 76.67%). This hypothesis should be verified in future studies, and it would be valuable if it would be addressed also in the context of patients with known pathogenic variants.

Robledo et al. found that MEN2A patients homozygous for the minor alleles of e11 rs1799939 (p.Gly691Ser) and e15 rs1800863 (p.Ser904=) are on average 10 years younger than carriers of other allele combinations [25]. In our study, the cluster characterised by the presence of minor alleles in those variants was cluster 3. The distribution of age at diagnosis was broader in cluster 3 than in cluster 2, and patients in cluster 3 seemed to have a younger age at diagnosis in general. Therefore, the trend was similar as in the cited paper. Our results were, however, not statistically significant. On the other hand, the observation in the study of Robledo et al. was statistically significant only for the homozygous state in non-sporadic MTC patients and other variants were not investigated in this study. It cannot be ruled out that this observation might possibly represent a broader situation in which the absence of those and other variants would be a factor protecting from earlier disease onset, as was seen in our study. Importantly, the age at diagnosis is only an approximation of the age of disease onset, and therefore, such results need to be interpreted carefully.

We observed an increased group of patients in the remission state in cluster 1. This cluster differed from clusters 2 and 3 by the presence of e13 rs1800861 (p.Leu769=) most of all, but also the rarer variant e14 rs1800862 (p.Ser836=), which was present in some patients of cluster 1 only. Importantly, none of those variants were significantly associated with remission when analysed independently. However, when e13 rs1800861 co-occurs with e14 rs1800862 and/or i14 rs2472737, a larger patient group might reveal a correlation between the likelihood to obtain a remission state of MTC in patients characterised by the presence of this combination.

For exploratory purposes, it would be interesting to verify whether the analysed variant combinations remain stable in thyroid tissues of patients with sporadic MTC. This would, however, not be feasible in routine clinical practice due to additional costs and technical issues related to working with tissue samples.

It should be noted that investigations have been performed that also include variants in other gene regions than the classically analysed exons 10, 11, and 13–16. In the Polish population in particular, the variant rs2435357 in intron 1 has been investigated, and results have shown that it might be advisable to consider their testing in addition to the variants included in our study [21,26]. Another question that might be interesting to address is to verify whether in any of the MTC patients, some of the common *RET* variants identified during diagnostic testing are in linkage disequilibrium with some rare disease-causing variants in untested regions. If confirmed, this might possibly help to understand the reasons underlying the observed effects.

We have not performed analyses in patients with pathogenic variants in the gene *RET*, as pathogenic variants influence the disease outcome to such a great extent that minor effects might have remained unidentified in a small sample of patients. However, our results might be of importance in the case of variants of unknown significance (VUS), where there is no consensus about their impact on the disease. On the basis of our results, it seems possible that the status of otherwise benign variants might influence disease outcome in patients with VUSes or even trigger disease occurrence. This hypothesis, however, needs to be verified in future. Another issue that seems to be worth addressing is whether germline benign or likely benign variants of the *RET* protooncogene impact the clinical outcome of sporadic MTC patients depending on somatic driver mutations in the tumour tissue, i.e., in *RET* and particularly *RAS* family protooncogenes, present in up to 80% of such cases [1].

## 4. Materials and Methods

### 4.1. Patients 

The study included patients from the south-eastern and south-central parts of Poland diagnosed with medullary thyroid carcinoma (MTC). Every patient with MTC had genetic testing of the gene *RET* performed as standard diagnostic procedure. The coding exons 10, 11, and 13–16 with intron-exon boundaries were analysed by Sanger sequencing on a 3500 Genetic Analyzer (Thermo Fisher, Waltham, MA, USA), using sequencing chemistry and sequencer consumables from Thermo Fisher. Additional exons in the gene were not evaluated as all participants were of Polish origin, given that no other exons have been confirmed to bear hot-spot regions for this population. For our analyses, only patients were chosen with no variants other than those studied in this work, i.e., rs1799939 in exon 11 (p.Gly691Ser), rs1800861 in exon 13 (p.Leu769=), rs1800862 in exon 14 (p.Ser836=), rs2472737 in intron 14, and rs1800863 in exon 15 (p.Ser904=). The list of patients selected for inclusion was further verified independently by two endocrinologists to ensure a proper diagnosis and the fulfilment of inclusion criteria. As a result, 48 MTC patients were included for analyses. The study group also contained patients with sporadic MTC but no *RET* variants (n = 5). As controls, 48 healthy volunteers were included, in whom thyroid disorders were excluded.

Inclusion criteria, MTC group: medullary thyroid carcinoma diagnosed at least two years before the beginning of the study; available molecular diagnostic result of the gene *RET*.

Exclusion criteria, MTC group: tumours other than MTC or signs and symptoms that may suggest multiple endocrine neoplasia type 2; familial history of MTC or MEN2 syndrome.

Inclusion criteria, control group: thyroid ultrasound negative for any pathological changes; euthyroidism; negative family history of thyroid disorders.

Exclusion criteria, control group: age below 30 years, which is justified by lowering the risk of a sporadic tumour appearing at a later age but simultaneously the need not to shift the age of controls away from the age of patients in the MTC group, in which younger individuals are also present.

In the MTC group, the following clinical parameters were analysed: age at diagnosis, MTC staging according to the American Joint Committee on Cancer Staging Manual 8th edition [18], remission of MTC, presence of the tumour relapse, TSH, carcinoembryonic antigen (CEA), calcitonin (Ct) and procalcitonin (proCt) concentrations, Ct/proCt ratio [1], co-occurrence of other tumours (MEN2 syndrome-related ones excluded), co-occurrence of metabolic diseases (dyslipidaemia, impaired fasting glycaemia, impaired glucose tolerance, diabetes mellitus type 2, gout or asymptomatic hyperuricaemia, metabolic syndrome), family history of other tumours in first-degree relatives, family history of metabolic diseases in first-degree relatives.

To identify minor effects driven by non-pathogenic *RET* variants, one must exclude large effects of pathogenic *RET* variants. Therefore, only patients with medullary thyroid carcinoma were included in the study, who were negative for variants in the gene *RET* that have been classified as pathogenic or of unknown significance according to NCBI database ClinVar and the Human Gene Mutation Database. In practice, only patients were included in the study who did not have any variants in the gene *RET* other than the five variants under investigation.

### 4.2. Genotyping

The molecular status of the following variants was confirmed by TaqMan analysis (Thermo Fisher, Waltham, MA, USA): rs1799939 in exon 11 (c.2071G>A; p.Gly691Ser), rs1800861 in exon 13 (c.2307G>T; p.Leu769=), rs1800862 in exon 14 (c.2508C>T; p.Ser836=), rs2472737 in intron 14 (c.2608-24G>A), and rs1800863 in exon 15 (c.2712C>G; p.Ser904=) of the gene *RET*. Briefly, DNA was isolated from whole peripheral blood collected into EDTA-containing tubes, by use of the NucleoSpin Blood kit (Macherey-Nagel, Dueren, Germany). Variants in the gene *RET* were discriminated using TaqMan SNP assays and the TaqMan Genotyping Master Mix from Thermo Fisher (Waltham, MA, USA), according to the manufacturer’s recommendations. TaqMan reads were performed on Mastercycler realplex2 (Eppendorf, Hamburg, Germany) and analysed using the incorporated software for data collection and analysis. Patients were assigned to be positive for a given variant if at least one copy of the rare allele was present in the patient.

### 4.3. Statistics

Minimal sample sizes were calculated in G*Power 3.1 software (Kiel, Germany) [27]. The analysis of haplotypes was performed in Haploview 4.2 (Cambridge, MA, USA) [28]. Hardy–Weinberg equilibrium was assessed by the genetics statistics tool for testing for deviation from Hardy–Weinberg equilibrium and tests for association from the Institute of Human Genetics, Technical University in Munich, Germany (https://ihg.gsf.de/ihg/snps.html (accessed on 8 October 2021)).

All remaining statistical analyses were performed in Statistica v13.0 (Tibco Software, Palo Alto, CA, USA). The significance cut-off value in all analyses was α = 0.05. Information on multiple comparison corrections is provided in the text.

For nominal variables, the CHI^2 test was used, with modifications where appropriate. For the comparison of continuous data between two groups, Student’s *t*-test was used for data with normal distribution and Mann–Whitney test for data with other than normal distribution. For the comparison of quantitative variables in more than two groups, Kruskal–Wallis analysis of variance was used, as all analysed data showed a distribution that was different than normal. In order to search for minimal allele combinations, we used stepwise logistic regression. For the generation of clusters with maximal genetic distance, unsupervised k-means clusterisation was implemented on the MTC group. In the case of CHI^2 tables larger than 2 × 2, correspondence analysis was used to visualise possible relations of nominal parameters.

### 4.4. Ethical Statement

The study has been approved by the Bioethics Committee of the Jagiellonian University in Krakow, Poland (opinion no. 1072.6120.207.2019). All study participants gave their informed consent for genetic analyses of the gene *RET* that were performed within the scope of the study.

## 5. Conclusions

Our data confirmed that common *RET* variants, identified during routine genetics diagnostics and classified clinically as benign, did not differ between MTC patients and healthy controls at a level that would be discriminative for the risk of MTC development. Our study is, therefore, in agreement with the main line of evidence for the benign clinical character of common variants in the gene *RET*.

However, some combined statuses of those variants might act as genetic modifiers and influence clinical parameters and/or the course of the disease in patients, in whom MTC occurs. The testing of such variants would come with no extra costs in laboratories that base the identification of *RET* variants on sequencing, as the *loci* of those variants are encompassed in standard genetic testing.

## Figures and Tables

**Table 1:** Minor allele frequencies.

Variant	MAF (%) Database ^1^	MAF (%) MTC Group	MAF (%) Control Group	*p* for MTC vs. Control Group
e11 rs1799939	18.47	17.71	15.63	0.6990
e13 rs1800861	23.32	28.13	34.38	0.3502
e14 rs1800862	4.90	3.13	3.13	1.0000
e15 rs1800863	18.58	18.75	15.63	0.5667
i14 rs2472737	22.56	16.67	19.79	0.5756

^1^ According to the Genome Aggregation Database (gnomAD) v2.1.1 for the European (non-Finnish) population.

**Table 2:** Fractions of haplotypes identified in the study population. Order of variants in the haplotypes: e11 rs1799939, e13 rs1800861, e14 rs1800862, e15 rs1800863, i14 rs2472737.

Haplotype	Control	MTC
GGCCG	0.312	0.250
GTCCG	0.302	0.365
GTCCA	0.198	0.167
ATCGG	0.156	0.177
GGTCG	0.031	0.031
GTCGG	0.000	0.010

**Table 3:** Comparison of clinical parameters between given variant carriers and sporadic MTC patients with no *RET* variant in the analysed regions. *p*-values for each analysis are shown.

Analysed Parameter	e11 rs1799939 *	e13 rs1800861	e14 rs1800862 *	e15 rs1800863 *	i14 rs2472737
Age at diagnosis	0.0682	0.0162	0.3687	0.1258	0.0809
Disease stage at diagnosis	0.6747	0.8680	0.9261	0.6055	0.5249
Lymph node metastases	0.5804	0.5879	1.0000	0.5804	1.0000
Distant metastases	1.0000	0.2445	1.0000	1.0000	1.0000
Relapses	0.7394	1.0000	0.5716	0.5761	1.0000
Remission state	0.5594	0.0879	0.1429	0.5594	0.1429
CEA concentrations	0.6879	0.8208	0.7656	0.6671	0.8345
Ct/proCt	0.5938	0.9511	0.3711	0.7595	0.4034
TSH concentrations	0.5934	0.1590	0.0736	0.8541	0.0216

* Some variants were not represented in an alone-standing setting. Therefore, e11 rs1799939 is represented by patients with this allele and e15 rs1800863; e15 rs1800863 is represented by patients with this allele only and patients with this allele together with e11 rs1799939; and e14 rs1800862 is represented by patients with this variant independently on the presence of other variants.

**Table 4:** Other disorders in the patients of the 3 clusters and their first-degree relatives, with no correction for multiple comparisons.

		Cluster 1	Cluster 2	Cluster 3	*p*-Value
Other tumours in the patients	No	11	6	11	0.5110
	Yes	7	4	3	
Metabolic disorders in the patients	No	17	6	10	0.0755
	Yes	1	4	4	
Tumours (other than MTC) in first-degree relatives	No	7	5	6	0.1578
	Yes	6	0	5	
Metabolic disorders in first-degree relatives	No	13	5	8	0.0647
	Yes	0	0	3	

## Data Availability

Data from Genome Aggregation Database (gnomAD) v2.1.1 have been used in this publication. Available at: https://gnomad.broadinstitute.org/ (accessed on 16 August 2021).
